# Chloroplast Protein Tic55 Involved in Dark-Induced Senescence through AtbHLH/AtWRKY-ANAC003 Controlling Pathway of *Arabidopsis thaliana*

**DOI:** 10.3390/genes13020308

**Published:** 2022-02-06

**Authors:** Chou-Yu Hsu, Ming-Lun Chou, Wan-Chen Wei, Yo-Chia Chung, Xin-Yue Loo, Lee-Fong Lin

**Affiliations:** 1 Department of Life Sciences, Tzu Chi University, Hualien 97004, Taiwan; 107711116@gms.tcu.edu.tw (C.-Y.H.); mlchou1015@gms.tcu.edu.tw (M.-L.C.); 107711102@gms.tcu.edu.tw (Y.-C.C.); 105711146@gms.tcu.edu.tw (X.-Y.L.); 2 Department of Surgery, Cheng-Ching Hospital, Chung-Kang Branch, Taichung 40764, Taiwan; wcwei0125@gmail.com

**Keywords:** chloroplasts Tic55 protein, dark-induced leaf senescence, *ANAC 003*, AtWRKY, AtbHLH transcription factors

## Abstract

The chloroplast comprises the outer and inner membranes that are composed of the translocon protein complexes Toc and Tic (translocon at the outer/inner envelope membrane of chloroplasts), respectively. Tic55, a chloroplast Tic protein member, was shown to be not vital for functional protein import in *Arabidopsis* from previous studies. Instead, Tic55 was revealed to be a dark-induced senescence-related protein in our earlier study. To explore whether Tic55 elicits other biological functions, a *tic55-II* knockout mutant (SALK_086048) was characterized under different stress treatments. Abiotic stress conditions, such as cold, heat, and high osmotic pressure, did not cause visible effects on *tic55-II* mutant plant, when compared to the wild type (WT). In contrast, senescence was induced in the individually darkened leaves (IDLs), resulting in the differential expression of the senescence-related genes *PEROXISOME DEFECTIVE 1* (*PED1*), *BLUE COPPER-BINDING PROTEIN* (*BCB*), *SENESCENCE 1* (*SEN1*), and *RUBISCO SMALL SUBUNIT GENE 2B* (*RBCS2B*). The absence of Tic55 in *tic55-II* knockout mutant inhibited expression of the senescence-related genes *PED1*, *BCB*, and *SEN1* at different stages of dark adaptation, while causing stimulation of *RBCS2B* gene expression at an early stage of dark response. Finally, yeast one-hybrid assays located the *ANAC003* promoter region with cis-acting elements are responsible for binding to the different AtbHLH proteins, thereby causing the transactivation of an *HIS3* reporter gene. ANAC003 was shown previously as a senescence-related protein and its activation would lead to expression of senescence-associated genes (SAGs), resulting in plant senescence. Thus, we propose a hypothetical model in which three signaling pathways may be involved in controlling the expression of *ANAC003*, followed by expression of SAGs that in turn leads to leaf senescence in *Arabidopsis* by this study and previous data.

## 1. Introduction

Chloroplasts are for the most part composed of proteins encoded by the nuclear genome and synthesized as precursor proteins (pre-proteins) in the cytosol, with the N-terminal transit peptide essential for targeting these pre-proteins to the appropriate membrane surfaces of chloroplasts. The N-terminal transit peptide of pre-proteins targeted to the chloroplasts via the Toc-Tic system is cleaved off by the stromal processing peptidase (SPP) and become mature proteins once pre-proteins enter the chloroplast stroma [1,2,3,4]. Several sub-components of the complex function as the channel/motor complex, Tic110, Tic40, and Hsp93; redox-regulatory subunits, Tic62, Tic55, and Tic32; as well as an alternative import channel, Tic20/Tic21 and Tic22 [5,6,7,8,9,10,11,12,13,14,15,16,17,18,19]. In addition to its possible role in chloroplast protein import, chloroplast inner membrane protein Tic55 may be involved in the senescence of *Arabidopsis*. It was reported previously that *Arabidopsis*-accelerated cell death gene *ACD1*, a potential *Pheophorbide a Oxygenase* (PaO)-encoding gene such as *Tic55*, is implicated in the senescence of *Arabidopsis* [19]. Based on the amino acid alignment and structure analysis among distinct species, Tic55 contains three highly conserved regions, including Rieske iron–sulfur motif, mononuclear iron-binding motif, and motif C (CxxC). Motif C is a thioredoxin binding motif and its biological role in Tic55 is still not clear in *Arabidopsis*. Furthermore, Tic55 was classified as one of the *LLS1*-related non-heme oxygenase gene family members, defined by the Rieske-type iron–sulfur center [20]. Other members in this family include *Lethal Leaf Spot 1* (*LLS1*)/ *Pheophorbide a Oxygenase* (*PAO*), *Chlorophyll a Oxygenase* (*CAO*), *Choline Monooxygenase* (*CMO*), and the precursor NADPH: protochlorophyllide oxidoreductase translocon protein (*Ptc52*). Several lines of evidence for *LLS1*-related non-heme oxygenases indicate that proteins in this family may play diverse roles in chlorophyll metabolism due to the photooxidative nature of photosynthesis [21]. Among these different functions, oxygenation of pheophorbide a by PAO elicits its important role in chlorophyll degradation. Pheophorbide a, an intermediate of chlorophyll degradation, recently was reported to regulate Jasmonate signaling during dark-induced senescence. Additionally, *Tic55*, *ACD1*, and *ACD1-like* genes are potential PaO-encoding genes [21]. Inhibition of *ACD1* gene expression should thus result in abnormal accumulation of pheophorbide a, an intermediate of the PAO/phyllobilin pathway, leading to rapid senescence of plants. However, no such significant effects were observed on pheophorbide a when *Tic55* gene expression was inhibited [21]. Tic55 in pea has been shown to play an important role in chloroplast pre-proteins import and redox motif is required to perform such function [3,16,17,18]. Recently, the biological function of Tic55 was identified as a hydroxylase of phyllobilins and may involve in plant senescence [21]. However, its direct relationship with plant senescence is still not clear even its indirect role is related to leaf senescence.

In order to dissect the potential direct biological function of Tic55 protein other than previously illustrated to be involved in the dark-induced aging [22], a *tic55* knockout mutant line (SALK_086048) obtained from the Arabidopsis Biological Resource Center (ABRC) was characterized and compared with wild type *Arabidopsis* (WT, Columbia) after several different stress treatments. The *tic55-II* mutant line was confirmed by both the genomic PCR and DNA sequencing for the precise site of T-DNA insertion. Individually darkened leaves (IDLs) experiments [23] showed the unique biological function of Tic55 as an aging-related protein. Further evidence of senescence-related gene expression, including *PED1*, *BCB*, *SEN1*, and *RBCS2B* in WT and *tic55-II* knockout mutant plants, supported the role of *Tic55* in senescence. Herein, our results are in agreement with our previous study in that the novel biological function of Tic55 is involved in the dark-induced senescence in *Arabidopsis* [22].

Leaf senescence is an intricate, highly coordinated, and likely to be orderly process. It requires integration of numerous internal and environmental signals [24,25]. Within the regulatory network, NAC, WRKY, and MYB TFs play central key roles in controlling transcriptional changes during senescence [26,27,28,29,30]. We have previously shown that MYB108 and its direct target gene *ANAC003* played a regulatory role in dark-stressed leaf senescence in *Arabidopsis thaliana* [22]. Several systematic analyses have provided valuable information regarding the distinct features and temporal transitions between the interactions of NAC transcription factors (TFs). For instance, time-series gene expression data sets of NAC TFs, presenting time-dependent networks, identified a shift from positive to negative regulation among NAC TFs [31,32]. In addition to NAC TFs, WRKY TFs function as positive regulators of leaf senescence. WRKY TFs are one of the largest families, functioning as transcriptional regulators and involved in signaling webs that modulate several plant processes. Recent researches reveal WRKY proteins often act as activators as well as repressors. Additionally, it is of interest to note that a single WRKY TF might be implicated in regulating several seemingly unrelated processes. While mechanisms of signaling and transcriptional regulation are being dissected, WRKY proteins are known to function via interactions with diverse protein partners, including MAP kinases, MAP kinase kinases, and other WRKY transcription factors [33]. Earlier studies have shown the expansion of WRKY family correlates well with the ongoing development of the sophisticated defense mechanism in land plants [34]. New findings reveal additional roles of WRKY proteins in germination, senescence, and responses to abiotic stresses such as drought and cold. The signature feature of WRKY TFs is their DNA binding domain, named WRKY domain which encompasses almost invariant WRKY amino acid sequence at the N-terminus of these proteins with only very few exceptions containing WRRY, WSKY, WKRY, WVKY, or WKKY instead [35]. On top of WRKY signature domain, WRKY TFs have atypical zinc-finger structure at the C-terminus. The zinc-finger structure is either Cx4-5Cx22-23HxH or Cx7Cx23HxC. The WRKY TFs are classified into three groups on the basis of the number of WRKY domains (two domains in Group I and one in the others) and the structure of their zinc fingers (C2HC in Group III proteins) [33]. WRKY6/45 promote leaf senescence by activating expression of senescence-associated genes (SAGs) and chlorophyll catabolic genes (CCGs), while WRKY46/51/63/75 trigger leaf senescence through the production of endogenous salicylic acid (SA). SA can induce natural leaf senescence [25,36]. Endogenous SA gradually increases when a leaf ages, causing the expression of many SAGs during leaf senescence. In addition, WRKY6, 22, 45, 54, 70, or 75, are thought to play crucial roles in leaf senescence [37,38,39,40,41]. Mitochondrial protease FtSH4 in *Arabidopsis* is involved in leaf senescence through regulation of WRKY-dependent signaling [41]. Knockdown or knockout of *WRKY45* or *WRKY75* delayed age-dependent leaf senescence, whereas overexpression of *WRKY45* or *WRKY75* resulted in acceleration of this process [39,40]. Besides WRKY TFs, basic helix–loop–helix (bHLH) domain-containing transcription factors are known for their roles in regulating various plant growth and developmental processes. For example, MdbHLH3 modulates leaf senescence in apple by promoting the expression of dehydratase—enolase–phosphatase complex 1 (MdDEP1) [42]. Our microarray data showed that several WRKY family transcripts, including At*WRKY31*, At*WRKY55*, At*WRKY72*, and At*WRKY75* were decreased noticeably in the *tic55-II-*knockout mutant with delayed dark-induced leaf senescence when compared to the WT *Arabidopsis*. From previous studies, we have found that the down-regulation of *ANAC003* was directly regulated by MYB108 [22]. To determine whether the decreased expression of *ANAC003*, *ANAC010*, *ANAC042*, and *ANAC075* in *tic55-II-*knockout mutant was controlled by WRKY TFs, a yeast one-hybrid assay was performed to detect whether the AtWRKYs can bind to the promoter of these aforesaid *ANACs*, respectively. In addition to the above-mentioned four different *AtWRKY* genes found down-regulated in our microarray results, four *AtbHLHs* (*AtbHLH19*, *AtbHLH41*, *AtbHLH92*, and *AtbHLH133*) were also down-regulated in *tic55-II* knockout mutant. Yeast one-hybrid analysis finally revealed that gene expression of *ANAC003*, a senescence-associated gene, could be regulated through at least three different pathways, including MYB108, AtWRKYs, and/or AtbHLHs-mediated networks, which in turn give rise to leaf senescence in *Arabidopsis*.

## 2. Materials and Methods

### 2.1. Plant Materials and Growth Conditions

All *Arabidopsis thaliana* plants utilized in this study were of the Columbia ecotype. The *tic40-2* mutant plants were obtained by screening T2 seeds from a T-DNA insertion population for seedling-lethal phenotypes (line 2490) and the T-DNA insertion position was determined by plasmid rescue [43]. Thus, *tic40-2* mutant plants were used as a kanamycin-resistant control in our screening. *Arabidopsis* plants were cultivated as described by Aronsson and Jarvis [17]. The T-DNA insertion mutant line for *AtTic55* (*At2g24820*), SALK_086048, was obtained from the Arabidopsis Biological Resource Center (ABRC) (www.biosci.ohio-state.edu/pcmb/Facilities/abrc/abrchome.htm) [44] and used in our studies. For the growth of plants, seeds were surface sterilized, sown on 1/2X Murashige and Skoog agar medium (1/2 MS medium) containing 2% (w/v) sucrose in Petri dishes, and kept in a growth chamber (22 °C, 16 h light/8 h dark). Throughout the paper, we have used 1/2 MS medium to grow all the *Arabidopsis* plants. To select for the T-DNA insertion mutants, kanamycin monosulfate with a final concentration of 50 µg/mL was added to the 1/2 MS medium. These *Arabidopsis* seedlings, on the other hand, were grown under a long-day cycle (16 h light/8 h dark) at 22 °C.

### 2.2. Genomic PCR and Semi-qRT-PCR

In order to obtain the homozygous mutant plants, *tic55-II-*knockout mutant plant (SALK_086048) was screened as described previously [22]. pGEM-T easy vector was subsequently used as a subcloning vector (Promega, Madison, WI, USA) for the genomic-PCR products. For the semi-quantitative RT-PCR (semi- qRT-PCR) assay, total RNA was isolated from the wild type plants and T-DNA insertion mutant (*tic55-II-*knockout mutant), respectively, and subjected to PCR reactions as described previously [22]. Briefly, the PCR reaction was carried out using primers: RT-F and RT-R (Table 1). The PCR products were then detected on agarose gels.

### 2.3. Stress Treatments

#### 2.3.1. Heat Stress Treatment

In total, four different heat exposures were carried out individually to determine the germination rate, root length, hypocotyl elongation, and survival rate, respectively, for both wild type (WT) and *tic55-II* knockout mutant. Roughly 20 seeds or seedlings were used for each analysis. In addition, these heat stress treatments were performed in seeds or seedlings according to the previous studies. For the first heat treatment to reveal the effect of high temperature on germination rate, seeds were heated at 45 °C for 4 h, cultured on 1/2 MS medium, and counted for germination rates [45]. In order to show the influence of heat stress on root length, 5-day/24 h light-grown seedlings were treated at 45 °C for 4 h, and then transferred back to grow under 24 h light condition. Six days later, the root length of each seedling was calculated and compared between WT and *tic55-II* knockout mutant [45]. To show the outcome of heat on hypocotyl elongation, 3-day/24 h dark-grown seedlings were consecutively treated to 38 °C for 2 h, 22 °C for 2 h, and then 45 °C for 2 h. Thereafter, these seedlings were moved back to 24 h dark condition for another three days. Hypocotyl elongation was then measured for both WT and *tic55-II* knockout mutant [45]. The survival rate was also measured after heat treatment for both WT and *tic55-II* knockout mutant. In this treatment, seedlings were grown under normal condition (16 h light/8 h dark) for 7 days, followed by exposing to 45 °C for 4 h, and then transported back to grow under normal condition. Seven days later, survival rates were determined.

#### 2.3.2. Cold Stress Treatment

Cold stress experiments were performed as described previously by Aronsson et al. [46]. Briefly, seedlings were grown under normal condition (16 h light/8 h dark) for 7 days and were then moved to 8 °C and cultivated for another 12 days under the same condition (16 h light/8 h dark). Thereafter, the phenotypes of both WT and *tic55-II* knockout mutant seedlings were assessed.

#### 2.3.3. High Osmotic Pressure Treatment

High osmotic pressure was created using mannitol as a solute in the 1/2 MS medium to cultivate Arabidopsis [47]. For this study, all plants were grown on 1/2 MS medium under 16 h light/8 h dark conditions and mannitol with a final concentration of 200 mM was added to the medium. Seven days later, both WT and tic55-II knockout mutant seedlings were assessed.

### 2.4. Dark Treatment and Senescence-Related Gene Expression by Semi qRT-PCR

#### 2.4.1. Dark-Induced Senescence

Based on studies by Wada et al. [23], the third to sixth rosette leaves of 19-day-old WT and *tic55-II* knockout mutant *Arabidopsis* plants were used for IDL treatment. The leaves were either covered with aluminum foils to prevent exposure to sunlight and grown for 5 days or left uncovered as untreated control. Five days later, both treated leaves (IDLs) and untreated (control leaves) were collected for WT and *tic55-II* plants. For these experiments, total RNA was extracted from leaves of roughly 30 seedlings of WT and *tic55-II* knockout mutant, respectively.

#### 2.4.2. Semi-Quantitative RT-PCR Assays

Total RNA from WT and mutant *tic55-II Arabidopsis* grown under normal or dark conditions was extracted and reverse transcribed using Goscript RT kit (Promega, Madison, WI, USA). The cDNA products were then used to perform PCR reactions using specific primer sets for each senescence-related gene of interest, followed by analysis by 1.5 % agarose gel electrophoresis. ACT2 was used as an internal control. The primers are given in Table 1.

#### 2.4.3. Chlorophyll Concentration Analysis

Quantitative analysis of chlorophyll a/b concentration was conducted based on the method of Porra [48]. In brief, the fifth and sixth leaves from normal and dark-treated 19-day-old seedlings were collected, weighed, frozen in liquid nitrogen, and stored at −80 °C. Next, the frozen leaves were ground in 80% acetone and centrifuged. Chlorophyll was extracted and collected from the supernatants by repeating the above steps until centrifuged pellets were almost completely white. Collected chlorophyll concentration was calculated by obtaining the absorbance at A663 and A647, respectively and then following the formulas: chlorophyll a (µg/mL) = (12.25 × A663) – (2.55 × A647); chlorophyll b (µg/mL) = (20.31 × A647) – (4.91 × A663); total chlorophyll (µg/mL) = (18.71 × A647) + (7.15 × A663) [47]. Finally, the chlorophyll concentrations were divided by the weight of leaf tissues and multiplied by the total collected volume.

### 2.5. Transactivation Assays in Yeast Cells

Different fusion constructs: pHIS2.1-*ANAC003p*, pHIS2.1-*ANAC010p*, pHIS2.1-*ANAC042p*, and pHIS2.1-*ANAC075p*, which carry distinct promoter region of *ANAC003*, *ANAC010*, *ANAC042*, or *ANAC075* gene, were established. Each promoter region comprises about 1 kb DNA fragment upstream of the transcriptional start site of the respective *ANAC* gene. In these pHIS2.1-*ANACp* fusion constructs, each *ANAC* promoter region is linked right before an *HIS3* reporter gene. In a similar fashion, plasmid pGADT7 containing an activation domain (AD) was employed to gain TF fusion constructs: pGADT7-*AtWRKY* (*AtWRKY31*, *AtWRKY55*, *AtWRKY72*, or *AtWRKY75*) and pGADT7-*AtbHLH* (*AtbHLH19*, *AtbHLH41*, *AtbHLH92*, or *AtbHLH133*). Each pGADT7 plasmid with *AtWRKY* or *AtbHLH* was then transformed together with one of the pHis2.1 plasmid encompassing discrete *ANAC* promoter (*ANAC003p*, *ANAC010p*, *ANAC042p*, or *ANAC075p*) into the AH109 yeast cells. In order to find the specific promoter region with cis-acting elements binding to either AtWRKY or AtbHLH proteins, full length (~ 1 kb upstream fragment of the various transcription factor gene), Δ1, or Δ2 promoter deletion mutants were transformed together with one of the diverse pGADT7-*AtWRKY* or pGADT7-*AtbHLH*, respectively. Thereafter, yeast one-hybrid analyses were carried out according to the instructions of Frozen-EZ yeast transformation II^TM^ (ZYMO Research, Orange, CA, USA). Thus, the transaction activity of each AtWRKY or AtbHLH protein was examined by the expression of the reporter *HIS3* gene. DNA [upstream activating sequences (UASs) and TATA boxes of the promoter region of *HIS3* reporter gene] and the protein (pGADT7-AtWRKY or pGADT7-AtbHLH fusion protein) interactions were determined by the growth conditions on the selective synthetic defined medium lacking leucine, tryptophan and histidine (SD/-Leu-Trp-His) as well as on the control (SD/-Leu-Trp) medium. DNA-protein interactions were finally determined by the spotting assays. All the spotting assays were at least repeated three times.

## 3. Results

### 3.1. Confirmation of tic55-II Knockout Mutant Line and Its Possible Biological Roles

**Figure 1:**
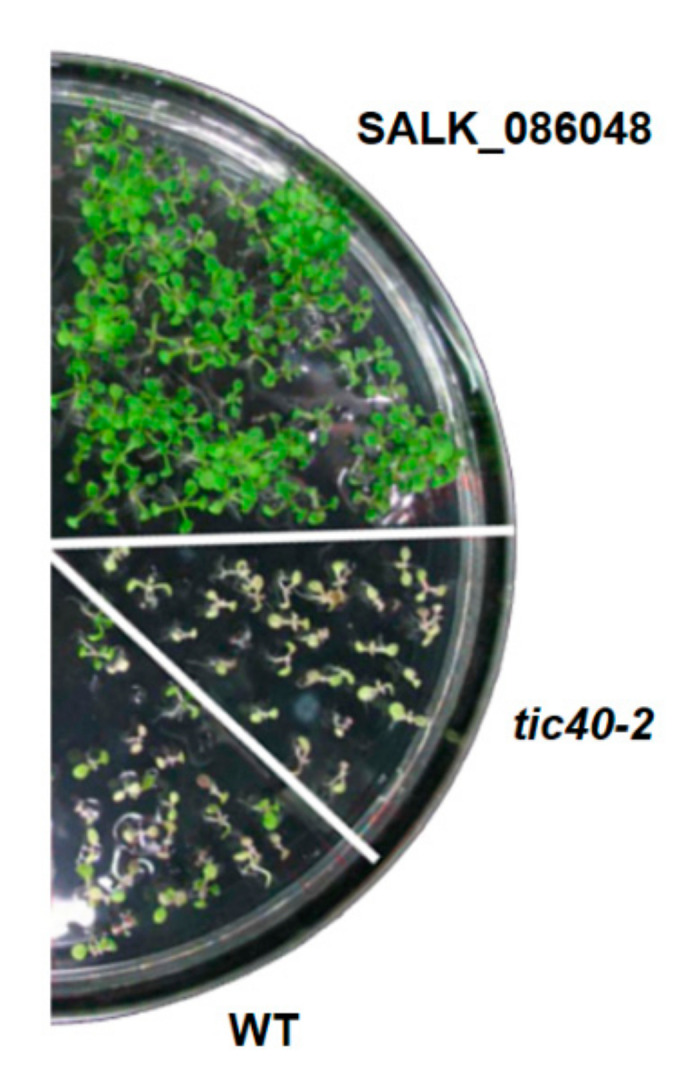
Confirmation of *Arabidopsis tic55-II* knockout mutant lines. Screening of homozygous mutants in 1/2 MS medium with kanamycin final concentration of 50 µg/mL. SALK_086048 represents *tic55-II* knockout mutant line obtained from the ARBC; WT for wild type plants (Col); while *tic40-2* plants contain a mutation in the inner membrane protein Tic40 that is not linked to antibiotic resistance, thus using as a kanamycin-resistant control. WT was used as a kanamycin-sensitive control.

In order to reveal the potential biological function other than dark-induced senescence of Tic55 in *Arabidopsis*, a *tic55-II* knockout line (SALK_086048) was obtained from the Arabidopsis Biological Resource Center (ABRC). As shown in Figure 1, the presence of the T-DNA insertion in this mutant did not affect the growth of *Arabidopsis*. In addition, this knockout mutant appeared to be homozygous individuals. From our previous data, we showed that Western blot analysis using Tic55 specific antibody (αTic55) further depicted the complete absence of Tic55 protein in chloroplast of this knockout line, named as *tic55-II*.

Based on previous studies, growth under extreme stress conditions may uncover the functions of certain proteins. For example, the ATP-driven import of the small subunit of Rubisco precursor (pRSS) into the plastids of pea was down-regulated by 67% and 49% in heat-stressed and chill-stressed plants, respectively [49]. In addition, heat stress of the *Arabidopsis wrky25* mutant decreased the germination rate and made shorter root systems and shorter hypocotyls [50]. Therefore, physical characterization of *tic55-II* knockout mutant under four distinct stress conditions was carried out in order to uncover the potential biological function of Tic55 protein other than previously illustrated to be involved in the dark-induced senescence [22]. No significantly different effects (*p* < 0.05) on germination rate, root length, hypocotyl elongation, and survival rate between the wild type (WT) and *tic55-II* knockout mutant (Figure S1A), suggesting a defect in Tic55 did not seem to affect their phenotype under heat stress conditions. Thus, Tic55 does not seem important in response to heat stress. In addition, cold stress experiments indicated no significant difference in phenotype between the WT and *tic55-II* knockout mutant after cold treatment (Figure S1B), both showing dark green and glassy phenotypes. Thus, there was no obvious change in cold responses in the absence of normal Tic55 protein. Since the inner plastid envelope membrane (IM), to which Tic55 is associated, is generally thought to be the osmo-regulatory barrier between the cytosol and the chloroplast stroma, high osmotic pressure was generated using mannitol as a solute in the MS medium which is used to cultivate *Arabidopsis* [47]. Mannitol, added to the medium to a final concentration of 200 mM, resulted in slower-growing and shorter plants (Figure S1C). Nevertheless, no significant phenotypic changes were observed between the WT and the *tic55-II* knockout mutant under high osmotic pressure, indicating that Tic55 is not crucial for the *Arabidopsis* osmotic response. Both temperature and light have significant effects on the chloroplast development and chlorophyll biosynthesis. Recent studies have reported that chill stress and heat stress impaired chlorophyll biosynthesis due to the down-regulation of gene expression and protein abundance of several enzymes implicated in the tetrapyrrole metabolism [51]. Furthermore, Aronsson et al. [52] showed that the generation of the Toc33 mutant *ppi1* was related to the intense light stress, resulting in the reduced chlorophyll production, when compared to the WT. Their data, therefore, suggested a decreased light-resistant response in the *ppi1* mutant. In this study, both WT and the *tic55-II* knockout mutant plants were exposed to the same intense light. The relative chlorophyll ratio of WT and knockout mutant *tic55-II* remained similar (Figure S1D). Clearly, chlorophyll biosynthesis in *tic55-II* knockout mutant was not significantly different from that of the wild type.

### 3.2. Tic55 Involved in the Chlorophyll Metabolism during Plant Senescence

**Figure 2:**
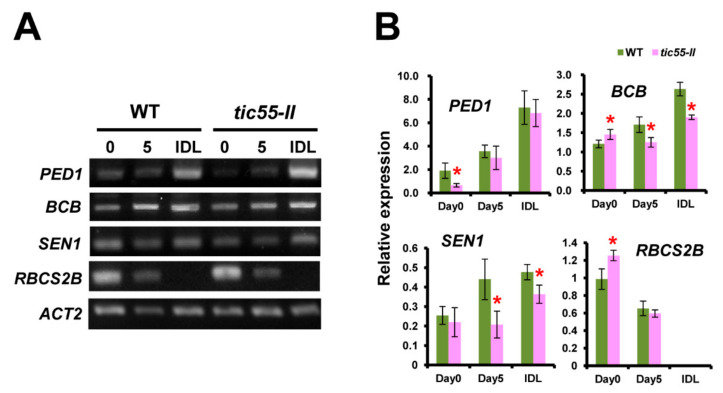
Dark-induced senescence and the importance of Tic55 in leaf senescence. (**A**) Semi-qRT-PCR analysis: Total RNA was extracted from the leaves of wild type (WT) and knockout mutant *tic55-II* on Day 0 or Day 5 without dark treatment (Control Day 0 and Control Day 5, respectively). Similarly, total RNA was extracted from the individually darkened leaves (IDLs) of WT and knockout mutant *tic55-II*, respectively, on Day 5 after dark treatment (IDL Day 5). The third and fourth rosette leaves of Day 0 plants were individually covered with foil soon after bolting, and the plants were grown for another 5 days before RNA was extracted. The senescence-related genes *PED1*, *BCB*, *SEN1*, and *RBCS2B* were reverse transcribed, followed by PCR for quantification. Semi-qRT-PCR products were analyzed by 1.5 % agarose gel electrophoresis. *ACT2* represents the internal control. (**B**) Relative senescence-related gene expression was determined quantitatively by using the *ACT2* as an internal control. Three independent analyses were conducted, and Student’s t-test was used to calculate the standard deviation. Green bars show the wild type *Arabidopsis* (WT), whereas pink bars represent *tic55-II* knockout mutant line. Asterisk (*) indicates *p* < 0.05.

Thus, the above-mentioned studies have failed to implicate Tic55 in temperature, osmosis, or light stress responses of *Arabidopsis* (Figure S1). It has been reported that dark-stimulated senescence in plants has a major effect on chlorophyll degradation [22]. Moreover, it is of interest to note that proteins similar to Tic55, in the LLS1-related non-heme family, are involved in the different stages of chlorophyll metabolism. For example, pheophorbide a oxygenase (PAO) and chlorophyllide a oxygenase (CAO) are implicated in chlorophyll degradation and chlorophyll biosynthesis, respectively [53,54]. Earlier studies also revealed that LLS1 suppressed cell death in maize cells [55]. Therefore, these data on none-heme family proteins suggested that Tic55 may play some role in the senescence of *Arabidopsis* [56]. To test this hypothesis, individually darkened leaves experiments (IDLs) [57] were conducted to induce senescence on soil-grown WT and *tic55-II* knockout mutant plants, respectively. Dark treatment was applied to the expanding third and fourth rosette leaves (Figure 2A: Control Day 0, Day 5, and IDL) when both WT and *tic55-II* knockout mutant plants were grown under long-day conditions (16 h light/8 h dark).

In addition, Tic55 contains a highly conserved (CxxC domain) thioredoxin target region, which may function in protein import and chlorophyll metabolism and is controlled by the light/dark and redox reactions [39]. Since Tic55 is not involved in the chloroplast protein import [19], but has a significant role in chlorophyll degradation during dark-induced senescence [22], we hypothesized that Tic55 may function through controlling thioredoxin in dark-induced senescence. To further investigate the degree of senescence as a result of dark treatment, expression levels of several senescence-related genes, namely *PEROXISOME DEFECTIVE 1* (*PED1*), *SENESCENCE 1* (*SEN1*), *BLUE COPPER-BINDING PROTEIN* (*BCB*), and *RUBISCO SMALL SUBUNIT GENE 2B* (*RBCS2B*) were analyzed. Previous studies showed that *PED1*, *SEN1*, and *BCB* were gradually up-regulated in response to plant senescence [23,58,59]. On the other hand, *RBC2B* gene expression was gradually inhibited as a result of senescence [23]. In this study, semi-quantitative RT-PCR (semi-qRT-PCR) of these four genes was carried out in order to determine the gene expression under normal and dark treatment within the WT and *tic55-II* knockout mutants (Figure 2A,B). At Day 5, the expression levels of *BCB* and *SEN1* were significantly lower in both untreated and dark-treated *tic55-II* knockout mutant plants compared to WT. Interestingly, *PED1* gene expression was significantly decreased prior to the dark treatment (Day 0) in the *tic55-II* knockout mutant (Figure 2B). These results are in accordance with our recent published data on chlorophyll concentrations measured in WT and *tic55-II* knockout mutant [22]. IDLs from *tic55-II* knockout mutant retained significantly more chlorophyll than the dark-treated WT at day 5. *RBCS2B* gene expression was significantly higher on Day 0 in the *tic55-II* knockout mutant. Decreased *RBCS2B* gene expression was noted in both untreated WT and *tic55-II* knockout mutants at Day 5 compared to Day 0, and no detectable *RBCS2B* gene expression in IDL for either line was observed (Figure 2B). Since the expression of senescence promoting genes is the opposite in *tic55-II* knockout mutants, the absence of Tic55 inhibited plant leaf senescence. These results thus revealed the relationship of Tic55 and senescence in the dark-induced senescence of *Arabidopsis*, which is in agreement with our previous data by investigating down-regulated expression of senescence-related genes of *tic55-II* knockout mutants based on microarray analysis (Table 2).

### 3.3. Possible TFs Regulate Plant Senescence

Based on our previous microarray data [22], both differential up- and down-regulated gene patterns were determined and their GO functional categories were assigned. Of the repressed transcripts in *tic55-II* mutant compared with wild type, two-fold change (*tic55-II*/WT) or greater in gene expression were selected for further investigation (Table 1). Namely three groups, including ANAC (NAM/ATAF1, 2/CUC2), AtWRKY, and AtbHLH family of transcription factors (TFs) in controlling the stress and senescence responses in *Arabidopsis* [12,17,28,29,30,39,40,49,51,54,55], are demonstrated in Table 2.

Additionally, transcription factors, such as WRKY, NAC, HSF, and bHLH families were responsive to dark-induced leaf senescence in Bermuda grass [60]. MdbHLH93 were shown to induce leaf senescence and the expression of senescence-associated gene MdSAG18 [61]. Thus, several lines of evidence revealed that WRKY and bHLH TFs may play vital roles in regulating leaf senescence. *ANAC003*, *ANAC010*, *ANAC042*, and *ANAC075* genes were found to be down-regulated in *tic55-II* knockout mutant in *Arabidopsis* based on microarray data (Table 2). *ANAC003* was further displayed to be involved in the dark-induced leaf senescence through MYB108 controlling pathway according to our previous data [22]. In this study, we identified potential AtWRKY and AtbHLH cis-acting elements (binding sites) in the promoter region of *ANAC003*, *ANAC010*, *ANAC042*, and *ANAC075*, respectively (Table 3 and Table 4), suggesting the expression of these *ANAC* genes maybe under the control of AtWRKY and/or AtbHLH TFs.

### 3.4. Yeast One-Hybrid Assays Showing Exoression of ANAC003 Is Regulated by AtWRKY and AtbHLH TFs, Respectively

**Figure 3:**
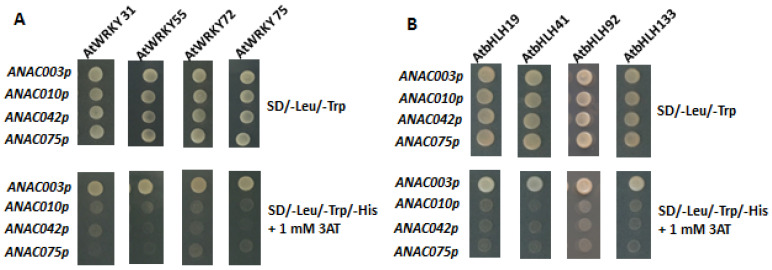

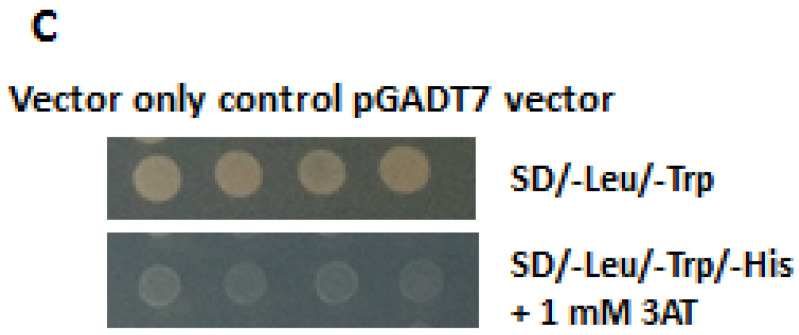
AtWRKY-ANAC and AtbHLH-ANAC relationships determined by yeast one-hybrid assays. Interaction between AtWRKY (AtWRKY31, AtWRKY55, AtWRKY72, or AtWRKY75) TF or AtbHLH (AtbHLH19, AtbHLH41, AtbHLH92, or AtbHLH133) TF and the promoters of different *ANACs* (*ANAC003p*, *ANAC010p*, *ANAC042p*, or *ANAC075p*) was analyzed. Promoter region (~ 1 kb upstream of the transcriptional start site) of the above-mentioned different *ANAC* genes was linked to an *HIS3* reporter gene, resulting in different constructs: pHIS2.1-*ANAC003p*, pHIS2.1-*ANAC010p*, pHIS2.1-*ANAC042p*, and pHIS2.1-*ANAC075p*. Each of these constructs was transformed into the yeast cells either with a plasmid carrying an Activation Domain (AD)-*AtWRKY* TF fusion (pGADT7-*AtWRKY*) or a pGADT7 plasmid with an *AtbHLH* TF (pGADT7-*AtbHLH*). (**A**) Yeast AH109 cells transformed with one of the different pGADT7-*AtWRKY* (*AtWRKY31*, *AtWRKY55*, *AtWRKY72*, or *AtWRKY75*) and one of the various pHis2.1-*ANACp* (*ANAC003p*, *ANAC010p*, *ANAC042p*, or *ANAC075p*) were cultured in the SD/-Leu/-Trp and SD/-Leu/-Trp/-His + 1 mM 3AT media, respectively. (**B**) Similarly, yeast cells transformed with one of the distinct pGADT7-*AtbHLH* (*AtbHLH19*, *AtbHLH41*, *AtbHLH92*, or *AtbHLH133*) and one of the diverse pHis2.1-*ANACp* (*ANAC003p*, *ANAC010p*, *ANAC042p*, or *ANAC075p*) were cultivated in the SD/-Leu/-Trp and SD/-Leu/-Trp/-His + 1 mM 3AT media, respectively. (**C**) pGADT7 vector only was transformed along with one of the various pHis2.1-*ANACp* (*ANAC003p*, *ANAC010p*, *ANAC042p*, or *ANAC075p*) into the yeast cells, which were then cultured in the SD/-Leu/-Trp and SD/-Leu/-Trp/-His + 1 mM 3AT media, respectively. Thus, pGADT7 vector only was used as a negative control in yeast one-hybrid assays.

In order to examine the transcription factor activity of AtWRKY and AtbHLH TFs, an *HIS3* reporter gene containing plasmid pHis2.1 was used to obtain different fusion constructs: pHIS2.1-*ANAC003p*, pHIS2.1-*ANAC010p*, pHIS2.1-*ANAC042p*, and pHIS2.1-*ANAC075p*, which carry distinct promoter region of *ANAC003*, *ANAC010*, *ANAC042*, or *ANAC075*. Each promoter region comprises about 1 kb DNA fragment upstream of the transcriptional start site of the respective *ANAC* gene. In a similar fashion, plasmid pGADT7 carrying an activation domain (AD) was employed to gain TF fusion constructs: pGADT7-*AtWRKY* (*AtWRKY31*, *AtWRKY55*, *AtWRKY72*, or *AtWRKY75*) and pGADT7-*AtbHLH* (*AtbHLH19*, *AtbHLH41*, *AtbHLH92*, or *AtbHLH133*). Each pGADT7 plasmid with *AtWRKY* or *AtbHLH* was then transformed together with pHis2.1 plasmid containing different *ANAC* promoter (*ANAC003p*, *ANAC010p*, *ANAC042p*, or *ANAC075p*) into the AH109 yeast cells. Thus, binding of AtWRKY TF or AtbHLH TF to the cis-acting elements of *ANAC003*, *ANA010*, *ANAC042*, or *ANAC075* promoter region would result in the expression of reporter *HIS3* gene. Herein, transformed yeast cells carrying both plasmids (pGADT7-*AtWRKY* or pGADT7-*AtbHLH*) and one of the pHIS2.1-*ANACp* grow in the SD/-Leu/-Trp/-His medium in the presence of 3-amino-1,2,4-triazol (3AT), indicating this specific TF (AtWRKY or AtbHLH) regulates the expression of *HIS3* reporter gene. The upper panel of Figure 3A exhibits that plasmids pGADT7-*AtWRKY* (*AtWRKY31*, *AtWRKY55*, *AtWRKY72*, or *AtWRKY75*) and pHIS2.1-*ANACp* (*ANAC003p*, *ANAC010p*, *ANAC042p*, or *ANAC075p*) were successfully transformed into the yeast cells, thereby growing in the SD/-Leu/-Trp media. The lower panel of Figure 3A shows that only promoter region of the *ANAC003* was bound by AtWRKYs, including AtWRKY31, AtWRKY55, AtWRKY72, or AtWRKY75, thereby causing the expression of *HIS3* reporter gene. Thus, these yeast cells grew in the SD/-Leu/-Trp/-His media in the presence of 3AT which was used to inhibit the basal expression of *HIS3* gene. Similarly, Figure 3B elicits that AtbHLHs, including AtbHLH19, AtbHLH41, AtbHLH92, and AtbHLH133, regulate only the expression of *ANAC003* gene as well. Vector pGADT7 only was used as a negative control, as shown in Figure 3C. Since no TF gene was present in the vector only control, no gene regulation was obtained when yeast cells were cultured in the SD/-Leu/-Trp/-His media, resulting in no growth of the yeast cells. Taken together, both AtWRKYs (AtWRKY31, AtWRKY55, AtWRKY72, and AtWRKY75) and AtbHLHs (AtbHLH19, AtbHLH41, AtbHLH92, and AtbHLH133) appeared to control the gene expression of *ANAC003* which was previously shown to be one of the senescence-associated genes in *Arabidopsis* [22]. From their studies, activated *ANAC003* gene expression would in turn activate the downstream expression of senescence associated genes (SAGs), resulting in the plant senescence. Thus, upregulated expression of both *AtWRKYs* (*AtWRKY31*, *AtWRKY55*, *AtWRKY72*, and *AtWRKY75*) and *AtbHLHs* (*AtbHLH19*, *AtbHLH41*, *AtbHLH92*, and *AtbHLH133*) in *tic55-II* knockout mutant when compared with wild type from our microarray data, suggesting that Tic55 is involved in the dark-induced senescence through AtbHLH/AtWRKY-ANAC003 controlling pathway.

### 3.5. Phylogenetic Analysis and Mutiple Sequence Alignment of AtWRKY and AtbHLH Proteins Related to Plant Senescence

**Figure 4:**
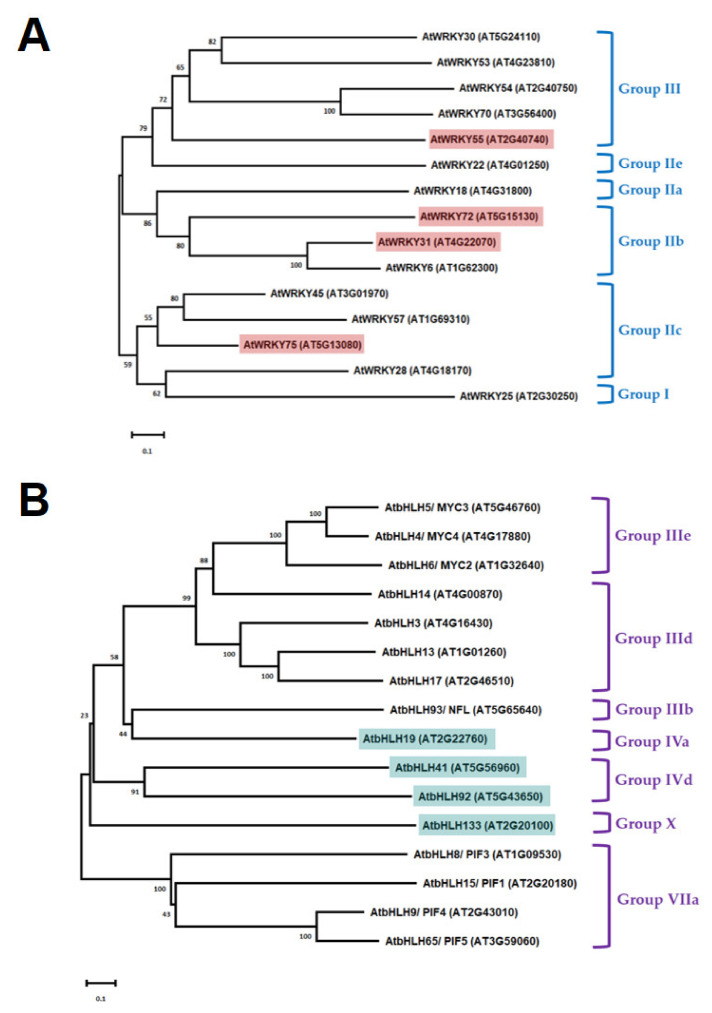
Phylogenetic analysis of different AtWRKY and AtbHLH proteins, respectively. (**A**) Phylogenetic relationships of 15 AtWRKY proteins, including AtWRKY31, AtWRKY55, AtWRKY72, and AtWRKY75, whose genes’ expression were down-regulated in *tic55-II* knockout mutant according to our microarray data (Table 2). Other AtWRKY proteins shown here are the senescence-related proteins in *Arabidopsis* [37,65,66,67]. (**B**) Phylogenetic tree of 16 AtbHLH proteins, including AtbHLH19, AtbHLH41, AtbHLH92, and AtbHLH133, which revealed down-regulated gene expression in *tic55-II* knockout mutant when compared with the wild type based on microarray analysis (Table 2). Other AtbHLH TFs were previously elicited to be senescence-associated proteins in *Arabidopsis* [29,68,69].

To explore the phylogenetic relationship of the AtWRKY proteins, a phylogenetic tree of 15 AtWRKY proteins, shown to be involved in plant senescence, was constructed by using the MEGA 6.0 [63] with the neighbor-joining method containing 1000 bootstrap replicates [64] (Figure 4A). Similarly, a phylogenetic tree composed of 16 AtbHLH proteins implicated in the plant senescence was established. Except for AtWRKY55, AtWRKY31, AtWRKY72, and AtWRKY75 belong to the same Group II, but different subgroups. AtWRKY55 is one of the members of Group III. On the other hand, AtbHLH19, AtbHLH41, and AtbHLH92 are the members of Group IV with AtbHLH133 belonging to the Group X (Figure 4B).

**Figure 5:**
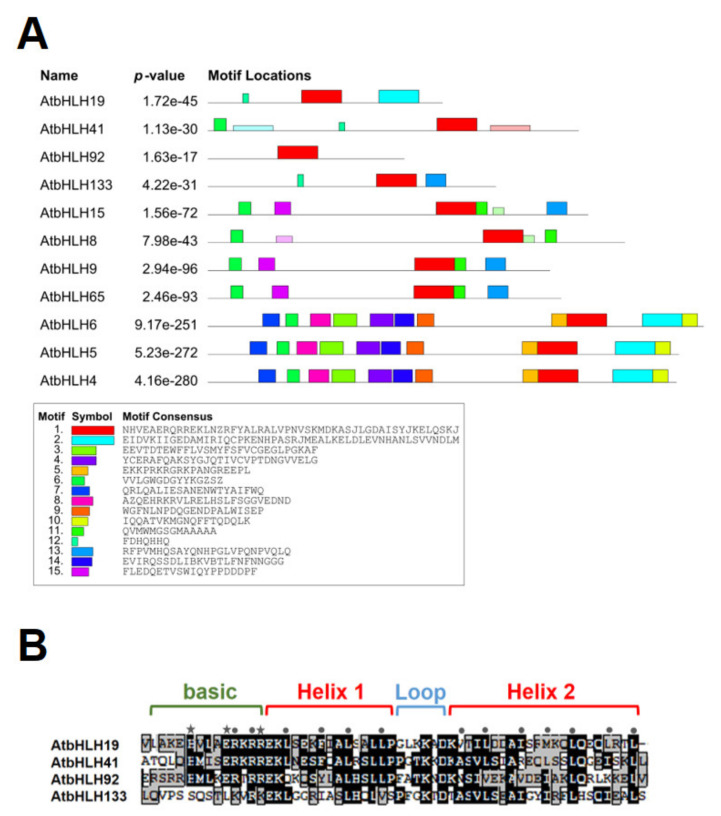
(**A**) MEME analysis of the senescence-related AtbHLH proteins. Red boxes represent the bHLH domain. All other different color boxes indicate the motifs around the red boxes, respectively. Lower panel shows the consensus sequences of the distinct motifs, including bHLH domain. (**B**) Amino acid alignment of the selected bHLH (basic Helix1–Loop–Helix2) domains of the AtbHLH19, AtbHLH41, AtbHLH92, and AtbHLH133. Black boxes elicit identical amino acid residues among different AtbHLH proteins, while gray boxes symbolize the amino acids with similar physical and chemical properties within these proteins.

The *AtbHLH* genes constitute one of the largest families of transcription factors in *A*. *thaliana*. There are twelve subfamilies that have been identified. By studying the *AtbHLH* genes entirely, there are conserved amino acid motifs outside the DNA binding domain within each of the main groups. In addition, gene redundancy among smaller subgroups was noticed [70]. Basically, a typical bHLH domain composed of a stretch of about 18 hydrophilic and basic amino acids at the N-terminus of the domain, followed by two regions of hydrophobic residues expected to generate amphipathic α-helices, separated by an intervening loop [71]. Domains other than the bHLH DNA binding domain, such as E12 factors of bHLH proteins in animals, revealed to be important for the regulation of gene expression by bHLH proteins [72]. The interaction of bHLH proteins within the same group or another group of TFs may also function in gene regulation. For example, recent research has shown that antagonism between bHLH subgroups, Group IIIe and IIId, modulates Jasmonate (JA)-induced leaf senescence in *Arabidopsis* [69]. In their study, subgroup IIIe, such as MYC2, MYC3, and MYC4, activates JA-induced leaf senescence, while subgroup IIId (bHLH3, bHLH13, bHLH14, and bHLH17) inhibits JA-induced leaf senescence, thereby regulating JA-induced senescence together. As shown in Figure 5A, multiple sequence alignment of the AtbHLH proteins, including AtbHLH19, AtbHLH41, AtbHLH92, AtbHLH133, and other senescence-related AtbHLH proteins, was analyzed according to the MEME analysis [72] and revealed all of them contain a bHLH domain (red boxes in Figure 5A). The consensus amino acid sequences of the bHLH domain and other motifs (shown in different colors) are exhibited in the lower panel of Figure 5A. Alignment of selected bHLH domains from AtbHLH19, AtbHLH41, AtbHLH92, and AtbHLH133 was illustrated in Figure 5B. There are amino acid residues important for DNA binding and/or protein–protein interaction. For example, asterisk indicates that these amino acids within the bHLH domain may contact with the nucleotide bases, while dots represent those may interact with DNA backbone. Pentagon shows nonpolar residues that may be involved in the protein–protein interaction [71], Figure 5B. One basic region and two α-helices are shown in Figure 5B. In addition, bHLH domain of the AtbHLH19, AtbHLH41, AtbHLH41, and AtbHLH133 contains a configuration of H-E-R with amino acid residues at positions 6, 10, and 14, respectively, which are marked with asterisks.

### 3.6. Location of the Binding Region with Cis-Acting Elements in the ANAC003 Promoter for Binding with Different AtbHLH Proteins

**Figure 6:**
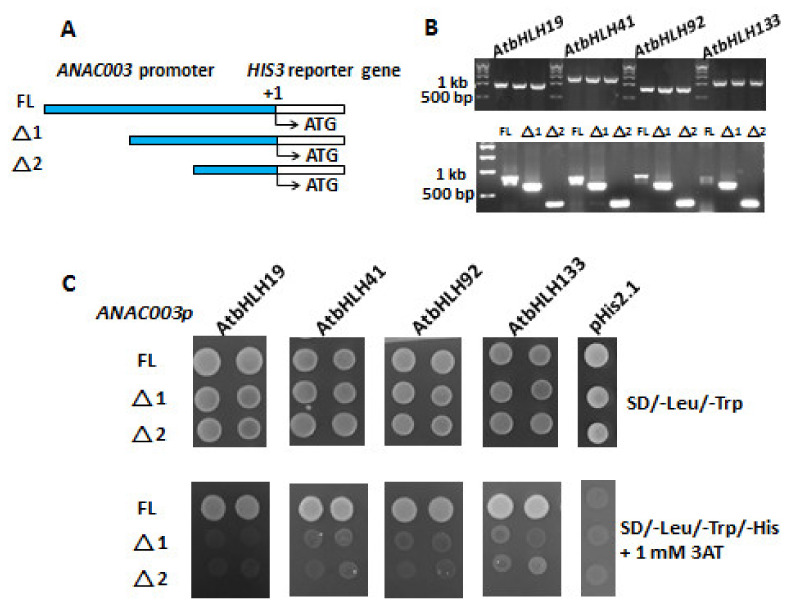
Transactivation assay for the location of cis-acting elements present in the *ANAC003* promoter. (**A**) Plasmid pHis2.1 vector carrying full length (FL) upstream promoter (about 1 kb), or two different promoter deletion mutants (Δ1 and Δ2) of *ANAC003* gene were constructed before a reporter gene (*HIS3*), respectively, as indicated. (**B**) Colony PCR results showed that pGADT7 vector containing At*bHLH19*, At*bHLH41*, At*bHLH92*, or At*bHLH133* gene were successfully transformed into the yeast AH109 cells, and therefore different At*bHLH* gene was amplified by using the specific primer set annealed to the pGADT7 vector (upper panel). Lower panel revealed that pHis2.1 vector encompassing FL, Δ1, or Δ2 promoter region of the *ANAC003* gene was amplified, respectively by using the primer set located at the pHis2.1 vector. (**C**) Yeast one-hybrid assay showing yeast cells with both pGADT7 and pHis2.1 vectors grew in the selection plates without amino acids leucine (Leu) and tryptophan (Trp). On the other hand, only yeast cells carrying pHis2.1 vector with full length (FL) promoter region (~1 kb) of the *ANAC003* gene grew in the absence of leucine (Leu), tryptophan (Trp), and histidine (His), indicating the reporter gene *HIS3* was activated and expressed by the transcription factors AtbHLH19, AtbHLH41, AtbHLH92, or AtbHLH133, respectively. Thus, yeast cells could grow in the selection plates without amino acid histidine (His). One mM of 3AT was used to inhibit the basal expression of the reporter gene. Plasmid pHis2.1 vector was employed as a negative control which did not carry any transcription factor gene.

In order to further explore the binding region containing cis-acting elements within the promoter region of the *ANAC003* gene for binding with the different AtbHLH proteins, we constructed distinct pHis2.1 vector carrying full length (FL, about 1 kb upstream of the *ANAC003* gene) and two different deletion mutants (Δ1 and Δ2) comprising discrete regions of the *ANAC003* promoter. As displayed in Figure 6A, FL, Δ1, and Δ2 of the *ANAC003* promoter located upstream of a reporter gene *HIS3* was shown. In order to examine the successful transformation of both recombinant DNAs containing a pHis2.1 vector with FL, Δ1, or Δ2 promoter region of the *ANAC003* gene and a pGADT7 vector with *AtbHLH19*, *AtbHLH41*, *AtbHLH92*, or *AtbHLH133* gene into the yeast strain AH109 cells, colony PCR assays were performed. Upper panel of Figure 6B revealed the amplified PCR fragments of the *AtbHLH19* (900 bps), *AtbHLH41* (1413 bps), *AtbHLH92* (756 bps), and *AtbHLH133* (1100 bps) genes, respectively, by using the specific primer set provided in Table 1. Similarly, lower panel of Figure 6B showed the PCR products of FL (800 bps), Δ1 (657 bps), and Δ2 (335 bps) of the promoter region of *ANAC003* gene, respectively, by using the specific primers listed in Table 1. Due to the primer set that was used, 800 bp fragment was amplified in FL, however, the actual length of the *ANAC003* promoter is 1 kb in FL. Nevertheless, Figure 6B elicited that vector pHis2.1 carrying FL, Δ1, or Δ2 and vector pGADT7 with *AtbHLH19*, *AtbHLH41*, *AtbHLH92*, or *AtbHLH133* gene were successfully transformed into yeast AH109 cells, respectively. Next, whether the expression of the *HIS3* reporter gene could be regulated by different AtbHLH proteins, including AtbHLH19, AtbHLH41, AtbHLH92, and AtbHLH133, was examined. Yeast one-hybrid assay was conducted by transforming different combination of various pHis2.1 encompassing distinct FL, Δ1, or Δ2 and pGADT7 with *AtbHLH19*, *AtbHLH41*, *AtbHLH92*, or *AtbHLH133* gene into the yeast cells. In total, 15 reactions including negative controls were performed. As shown in the upper panel of Figure 6C, all the yeast cells grew in the selection plates without the presence of the amino acids Leu and Trp, meaning plasmids pHis2.1 with different length of *ANAC003* promoter (FL, Δ1, or Δ2) and pGADT7 with various *AtbHLH* genes (*AtbHLH19*, *AtbHLH41*, *AtbHLH92*, or *AtbHLH133*) were transformed successfully into the yeast cells. Vector pHis2.1 alone was employed as a negative control. In the lower panel of Figure 6C exhibited the major results of this yeast one-hybrid assay in which the expression of the reporter *HIS3* gene was activated in the presence of a pHis2.1 vector enclosing the full length (FL) of the *ANAC003* promoter (~1 kb upstream of the *ANAC003* gene), resulting in the growth of the transformed yeast cells in the absence of the amino acids Leu, Trp, and His. On the contrary, expression of *HIS3* gene was not obtained in pHis2.1−Δ1, or pHis2.1−Δ2 constructs, leading to no growth of the transformed yeast cells in the plates without amino acids Leu, Trp, and His. Thus, these data indicated that the cis-acting elements are present in the upstream region before the Δ1 promoter fragment.

## 4. Discussion

It had been demonstrated that Tic55 associated with several translocon proteins, such as Tic32, Tic62, Tic110, and Tic40, that locate to the inner envelope membrane of the chloroplast in pea and function in chloroplast protein import [8,16,17]. In contrast to pea Tic55, our previous study revealed that Tic55 plays an important role in dark-induced senescence in *Arabidopsis* [22]. As characterized previously [22], the homozygous *tic55-II* knockout mutant showed no significant phenotypic differences between the wild type *Arabidopsis* and *tic55-II* knockout mutant, suggesting that Tic55 may not be functionally important to the survival of *Arabidopsis thaliana*. A unique biological function of Tic55 was finally revealed when *Arabidopsis* was aged under dark treatment (IDLs) [57]. To further investigate whether Tic55 may be involved in other important biological functions, both wild type and the *tic55-II* knockout mutant were exposed to different stresses since that would be an effective method to explore the potential biological activities of interested proteins. Unfortunately, distinct stress conditions did not cause any phenotypic changes between the wild type and the knockout mutant *tic55-II*, suggesting that no other biological function was found so far for Tic55 other than involved in the dark-induced senescence (Figure S1). Senescence-related gene expression correlated with the presence of Tic55 function (Figure 2). The absence of Tic55 in *tic55-II* knockout mutant inhibited expression of the senescence-related genes *PED1*, *BCB*, and *SEN1* at different stages of dark adaptation, and stimulation of *RBCS2B* gene expression appeared at an early stage of dark response. Our data thus showed that the novel biological function of Tic55 is only related to the dark-induced senescence of *Arabidopsis*.

**Figure 7:**
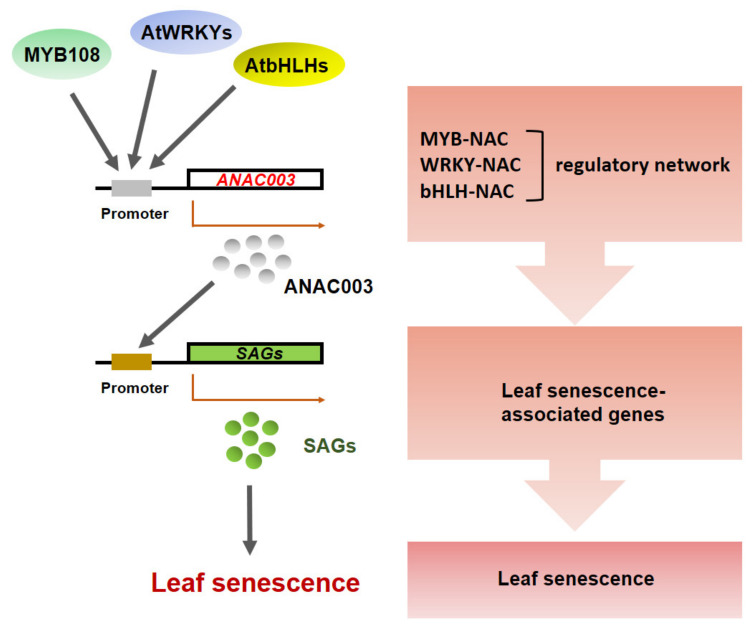
Hypothetical model showing AtbHLH/AtWRKY/MYB108-ANAC003 controlling pathways involved in the regulation of leaf senescence in *Arabidopsis*. MYB108, AtWRKYs, AtbHLHs represent members of the different transcription factor families. ANAC003 is one of members of ANAC transcription factor family. SAGs are short for senescence-associated genes.

According to our previous microarray results [22], we selected those with at least two-fold down-regulated expression of genes, including four *ANACs* (*ANAC003*, *ANAC010*, *ANAC042*, and *ANAC075*), four *AtWRKYs* (*AtWRKY31*, *AtWRKY55*, *AtWRKY72*, and *AtWRKY75*), and four *AtbHLHs* (*AtbHLH19*, *AtbHLH41*, *AtbHLH92*, and *AtbHLH133*), belonging to three transcription factor families, respectively, in *tic55-II* knockout mutant for further investigation. Our earlier studies revealed that gene expression of *ANAC003* is activated by MYB108 transcription factor [22], and *ANAC003* is a senescence-associated gene whose expression contributes to the plant senescence [28,32]. In this study, we identified the potential binding sites (cis-acting elements) located within the promoter regions of the various *ANACs* genes, including *ANAC003*, *ANAC010*, *ANAC042*, and *ANAC075*, by using the PlantPAN 2.0 program [62] (Table 3 and Table 4). Although all the promoters of these four *ANACs* appeared to contain the potential cis-acting elements for binding with four different AtWRKYs and four AtbHLHs, respectively, yeast one-hybrid assay showed only the promoter region of *ANAC003* was bound by distinct AtWRKYs and AtbHLHs, respectively, as shown in Figure 3. These data thus indicate that the *ANAC003* gene expression could be regulated by either AtWRKY (AtWRKY31, AtWRKY55, AtWRKY72, or AtWRKY75) or AtbHLH (AtbHLH19, AtbHLH41, AtbHLH92, or AtbHLH133) proteins. Subsequently, we analyzed the phylogenetic relationship of the above-mentioned four AtWRKYs involved in *ANAC003* gene regulation with other AtWRKYs that were known to be related to plant senescence. Similarly, the four aforesaid AtbHLHs of this study with other AtbHLHs important in the regulation of plant senescence were examined as well (Figure 4). Our data represented in this study thus revealed several potential regulatory networks implicated in the regulation of senescence-associated gene *ANAC003* expression. The structures of AtbHLH proteins containing several conserved motifs along with a bHLH domain, which is composed of a stretch of hydrophilic and basic amino acids at the N-terminus of the domain, an intervening loop, followed by two regions expected to form amphipathic α-helices, were identified [70,71,72,73]. As shown in Figure 5, all the AtbHLH proteins contain a conserved bHLH domain and the amino acid sequences of this bHLH domain were further closely described in Figure 5B. Finally, two distinct deletion mutants encompassing discrete segments of the promoter region of *ANAC003* gene were constructed in order to locate the region with cis-acting elements that are responsible for binding to different AtbHLH proteins, thereby resulting in the transactivation of an *HIS3* reporter gene. These mutants were compared with that of the full-length promoter (~1 kb upstream of the transcriptional start site of the *ANAC003* gene) in gene regulation of the *HIS3* reporter gene. Our results concluded that the region containing the upstream sequences before Δ1 deletion mutant appeared to be important in binding distinct transcription factors, including AtbHLH19, AtbHLH41, AtbHLH92, and AtbHLH133, thereby causing the transactivation of *ANAC003* gene expression. Thus, we propose that chloroplast protein Tic55 may be involved in the AtbHLH/AtWRKY/MYB108-ANAC003 controlling pathway during dark-induced senescence in *Arabidopsis*, as shown in Figure 7.

## 5. Conclusions

In brief, the chloroplast comprises the outer and inner membranes which are composed of the translocon protein complexes Toc and Tic, respectively. Tic55, a chloroplast Tic protein member, was shown not vital for functional protein import in *Arabidopsis* from previous studies. Instead, Tic55 revealed to be a dark-induced senescence-related protein in our earlier study [22]. To explore whether Tic55 elicits other biological functions, a *tic55-II* knockout mutant (SALK_086048) was characterized under different stress conditions, such as cold, heat, and high osmotic pressure, did not cause visible effects on *tic55-II* mutant plant, when compared to the wild type (WT). In contrast, senescence was induced in the individually darkened leaves (IDLs), resulting in the differential expression of the senescence-related genes *PEROXISOME DEFECTIVE 1* (*PED1*), *BLUE COPPER-BINDING PROTEIN* (*BCB*), *SENESCENCE 1* (*SEN1*), and *RUBISCO SMALL SUBUNIT GENE 2B* (*RBCS2B*). The absence of Tic55 in *tic55-II* knockout mutant inhibited expression of the senescence-related genes *PED1*, *BCB*, and *SEN1* at different stages of dark adaptation, while causing stimulation of *RBCS2B* gene expression at an early stage of dark response. Finally, yeast one-hybrid assays located the *ANAC003* promoter region with cis-acting elements that are responsible for binding to the different AtbHLH proteins, thereby resulting in the transactivation of a *HIS3* reporter gene. Thus, we propose a hypothetical model in which three signaling pathways may be involved in controlling the expression of *ANAC003*, followed by expression of senescence-associated genes (SAGs) which in turn leads to leaf senescence in *Arabidopsis* by this study and our previous data.

## Figures and Tables

**Table 1:** Primers used in this study.

Target Genes	Primer Name	Oligo Sequence (5′ to 3′)	Purpose
*PED1*	PED1-RT-F	GCAATCATGGGTATCGGTCCAG	Semi qRT-PCR
	PED1-RT-R	CTAGCGAGCGTCCTTGGACAAA	Semi qRT-PCR
*SEN1*	SEN1-RT-F	ATCACGAATTGGAAACTGG	Semi qRT-PCR
	SEN1-RT-R	CTTTCCTCCATCGGAAG	Semi qRT-PCR
*BCB*	BCB-RT-F	GAGGCATGATGTGGCAGTTGTAT	Semi qRT-PCR
	BCB-RT-R	TCAAAAGAGAGCAACAACAGCAG	Semi qRT-PCR
*RBCS2B*	RBCS2B-RT-F	ACCTTCTCCGCAACAAGTGG	Semi qRT-PCR
	RBCS2B-RT-R	GAAGCTTGGTGGCTTGTAGG	Semi qRT-PCR
*Actin2*	ACT2-RT-F	ACATTGCAAAGAGTTTCAAGGT	Semi qRT-PCR
	ACT2-RT-R	CTAAGCTCTCAAGATCAAAGG	Semi qRT-PCR
*AtbHLH19*	pGADT7-F	TAATACGACTCACTATAGGGC	Colony PCR
*(AtbHLH41*, *AtbHLH92*, *AtbHLH133)*	pGADT7-R	AGATGGTGCACGATGCACAG	Colony PCR
*ANAC003p*	pHis2.1-F	CTATAGGGCGAATTCGAG	Colony PCR
*(*FL, △1, △2*)*	pHis2.1-R	GATAATGCCAGGAATTACTAG	Colony PCR

**Table 2:** List of significantly downregulated ANAC, AtWRKY, and AtbHLH genes subcategories from the biological processes categories according to the gene ontology classification scheme from microarray analysis. Data provided represent fold expression Log_2_ (*tic55-II*-knockout/wild type).

Locus ID	GO Term	Annotation	Expression	Fold Change (*tic55-II*/WT)
NM_100103	GO:0010228~vegetative to reproductive phase transition of meristem GO:0003700~transcription factor activity GO:0016926~ protein desumoylation GO:0007275~multicellular organism development GO:0005634~ nucleus GO:0050665~hydrogen peroxide biosynthetic process	*A. thaliana* NAC domain containing protein 3 mRNA (*ANAC003*)	Down	−2.237
NM_102615	GO:0010228~ vegetative to reproductive phase transition of meristem GO:0003700~ transcription factor activity GO:2000652~ regulation of secondary cell wall biogenesis GO:0016926~ protein desumoylation GO:0007275~ multicellular organism development GO:0005634~ nucleus GO:0050665~ hydrogen peroxide biosynthetic process GO:0045893~ positive regulation of transcription, DNA-templated	*A. thaliana* NAC domain containing protein 10 mRNA (*ANAC010*)	Down	−5.360
NM_129861	GO:1900056~ negative regulation of leaf senescence GO:0034976~ response to endoplasmic reticulum stress GO:0003700~ transcription factor activity GO:0009718~ anthocyanin- containing compound biosynthetic process GO:0005634~ nucleus GO:0007275~ multicellular organism development GO:0042538~ hyperosmotic salinity response GO:0010120~ camalexin biosynthetic process GO:0010150~ leaf senescence GO:0009627~systemic acquired resistance GO:0005992~ trehalose biosynthetic process GO:0006561~ proline biosynthetic process GO:0009723~ response to ethylene	*A. thaliana* NAC domain containing protein 42 mRNA (*ANAC042*)	Down	−2.244
NM_119067	GO:0003700~transcription factor activity GO:0007275~multicellular organism development GO:0005634~ nucleus	*A. thaliana* NAC domain containing protein 75 mRNA (*ANAC075*)	Down	−3.109
NM_118328	GO:0003677~ DNA binding GO:0003700~ transcription factor activity GO:0030528~ transcription regulator activity GO:0043565~ sequence-specific DNA binding	*A. thaliana* WRKY DNA-binding protein 31 mRNA (*AtWRKY31*)	Down	−3.310
NM_001084564	GO:0003677~ DNA binding GO:0003700~ transcription factor activity GO:0030528~ transcription regulator activity GO:0043565~ sequence-specific DNA binding	*A. thaliana* WRKY transcription factor 55 mRNA (*AtWRKY55*)	Down	−2.839
NM_121517	GO:0003677~ DNA binding GO:0003700~ transcription factor activity GO:0030528~ transcription regulator activity GO:0043565~ sequence-specific DNA binding	*A. thaliana* putative WRKY transcription factor 72 mRNA (*AtWRKY72*)	Down	−7.990
NM_121311	GO:0003677~ DNA binding GO:0003700~ transcription factor activity GO:0030528~ transcription regulator activity GO:0043565~ sequence-specific DNA binding	*A. thaliana* putative WRKY transcription factor 75 mRNA (*AtWRKY75*)	Down	−2.627
NM_127841	GO:0003677~ DNA binding GO:0003700~ transcription factor activity GO:005634~ nucleus GO:006355~ regulation of transcription, DNA-templated GO:0010200~ response to chitin	*A. thaliana* transcription factor bHLH19 mRNA (*AtbHLH19*)	Down	−3.046
NM_125078	GO:0003677~ DNA binding GO:0003700~ transcription factor activity GO:005634~ nucleus GO:006355~ regulation of transcription, DNA-templated	*A. thaliana* transcription factor bHLH41 mRNA (*AtbHLH41*)	Down	−3.677
NM_123731	GO:0003677~ DNA binding GO:0003700~ transcription factor activity GO:005634~ nucleus	*A. thaliana* transcription factor bHLH92 mRNA (*AtbHLH92*)	Down	−4.638
NM_127568	GO:005634~ Nucleus	*A. thaliana* transcription factor bHLH133 mRNA (*AtbHLH133*)	Down	−2.626

**Table 3:** Potential AtWRKY binding sites predicted by PlantPAN 2.0 analyses [62].

NAC Genes	AtWRKY Binding Sequences (Nucleotides No.) ^1^
*ANAC003*	TGACT (−207~−203); TGACT (−399~−403); TTGACA (−755~−750) TTGACC (−876~-881); TTGACG (-949~-954)
*ANAC010*	TGACC (−32~−36); TTGACG (−222~−217); TGACT (−294~−298) TTGACA (−927~−932)
*ANAC042*	TGACT (−39~−35); TTGACC (−130~-125); TTTGACT (−143~−149) TGACT (−789~−785)
*ANAC075*	TGACC (−623~−627)

^1^ Nucleotide sequence numbers correspond to the sequences upstream of the transcriptional start site of their respective NAC genes.

**Table 4:** Potential AtbHLH binding sites predicted by PlantPAN 2.0 analyses [62].

NAC Genes	AtbHLH Binding Sequences (Nucleotides No.) ^1^
*ANAC003*	AGACGTAT (−113~−119); ATACTTGT (−216~−222); ATACGTGG (−279~−286); CAAGTG (−305~−300); ACACGTAA (−524~−531); CAAATG (−599~−594); ATACTTTT (−717~−710); GCAAGTTC (−780~−773); CAAATGCATATG (−785~−796); CAAATG (−824~−829); CAATTG (−833~−838); ACCAGT (−855~−860); ATTCGTGG (−873~−866); ATATGAGT (−875~−882); CATATG (−888~−893); TTACGTTT (−972~−965)
*ANAC010*	CAAGTG (−28~−33); CAAGTG (−124~−129); ATACGTTT (−244~−251); CAACTG (−343~−338); CATATG (−613~−608); ATACATGA (−748~-741); ACCAAGTTGGT (−839~−829); CACGAG (−882~−877)
*ANAC042*	TGACT (−39~−35); TTGACC (−130~−125); TTTGACT (−143~−149) TGACT (−789~−785)
*ANAC075*	TGACC (−623~−627)

^1^ Nucleotide sequence numbers correspond to the sequences upstream of the transcriptional start site of their respective NAC genes.

## Data Availability

Not applicable.

## Supplementary Materials

The following are available online at https://www.mdpi.com/article/10.3390/genes13020308/s1, Figure S1: Physical characterization of *tic55-II* mutant compared to the wild type (WT) under different stress conditions.
