# Supplementary material for: Chloroplast Protein Tic55 Involved in Dark-Induced Senescence through AtbHLH/AtWRKY-ANAC003 Controlling Pathway of Arabidopsis thaliana
Source: Genes (Basel). 2022 Feb 6;13(2):308. doi: 10.3390/genes13020308 (PMC8872272; doi:10.3390/genes13020308)
Supplement: Supplementary file 1 [file genes-13-00308-s001.zip › genes-1550496-supplementary.pdf]

genes

Article

Chloroplast Protein Tic55 Involved in Dark-Induced Senescence through AtbHLH/AtWRKY-ANAC003 Controlling Pathway of *Arabidopsis thaliana*

Chou-Yu Hsu [1,+], Ming-Lun Chou [1,+], Wan-Chen Wei [2], Yo-Chia Chung [1], Xin-Yue Loo [1], and Lee-Fong Lin [1,*]

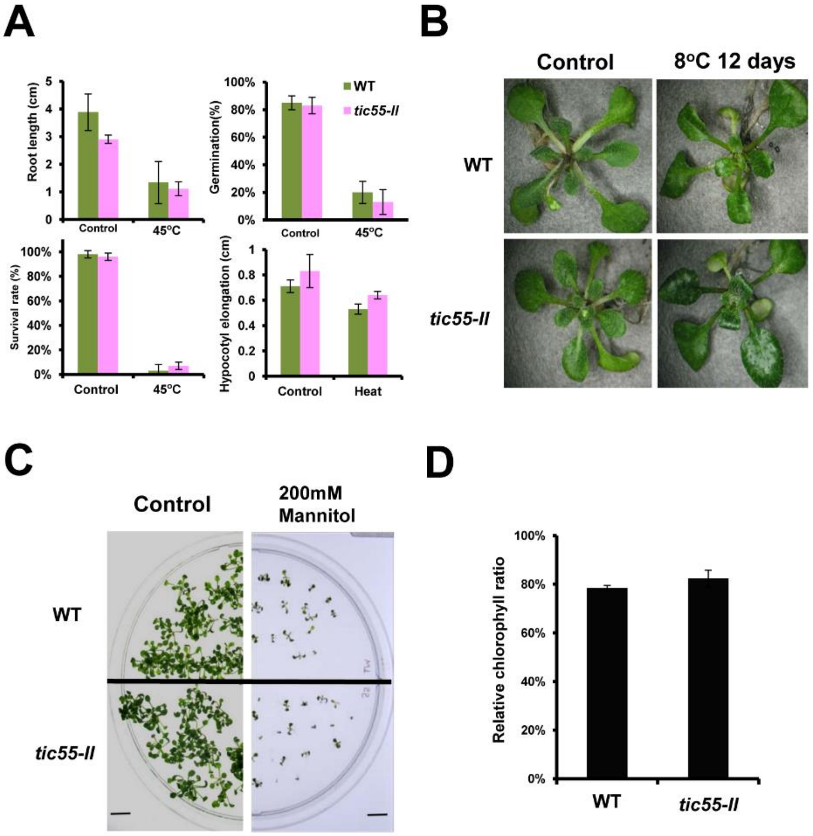

Figure S1. Physical characterization of *tic55-II* mutant compared to the wild type (WT) under different stress conditions. (**A**) Germination rates (%), root length (cm), hypocotyl elongation (cm), and survival rates (%) for both WT (control) and knockout mutant *tic55-II* seedlings were determined quantitatively under heat stress condition. Green bars represent WT, while pink bars indicate knockout mutant *tic55-II*. Three independent experiments were conducted and standard deviation was calculated. (**B**) Under chill-stress situation, the phenotypic appearance of the knockout mutant *tic55-II* and WT seedlings was observed. Control indicates normal growth condition (22 ^{0}C, 16 h light/8 h dark). (**C**) High osmotic stress condition was generated by using mannitol with a final concentration of 200 mM added in the culture medium (1/2 MS medium). Germination for WT and knockout mutant *tic55-II* was observed. Control represents seeds cultured in the absence of mannitol. Scale bars indicate 1 cm. (**D**) The effects of light on Chlorophyll biogenesis was determined for WT and knockout mutant line *tic55-II*, respectively, under intense light exposure (147.17μmol/m^2/s), in relation to the normal light treatment (113.21μmol/m^2/s). Relative chlorophyll ratio

was calculated quantitatively as the amounts of chlorophyll in mutant line normalized by those of the wild type. Three independent assays were carried out and standard deviation was determined.
